# Steering Endogenous Butyrate Production in the Intestinal Tract of Broilers as a Tool to Improve Gut Health

**DOI:** 10.3389/fvets.2015.00075

**Published:** 2015-12-17

**Authors:** Lonneke Onrust, Richard Ducatelle, Karolien Van Driessche, Celine De Maesschalck, Karen Vermeulen, Freddy Haesebrouck, Venessa Eeckhaut, Filip Van Immerseel

**Affiliations:** ^1^Department of Pathology, Bacteriology and Avian Diseases, Ghent University, Merelbeke, Belgium

**Keywords:** broiler, endogenous butyrate, performance, probiotics, prebiotics

## Abstract

The ban on antimicrobial growth promoters and efforts to reduce therapeutic antibiotic usage has led to major problems of gastrointestinal dysbiosis in livestock production in Europe. Control of dysbiosis without the use of antibiotics requires a thorough understanding of the interaction between the microbiota and the host mucosa. The gut microbiota of the healthy chicken is highly diverse, producing various metabolic end products, including gases and fermentation acids. The distal gut knows an abundance of bacteria from within the *Firmicutes Clostridium* clusters IV and XIVa that produce butyric acid, which is one of the metabolites that are sensed by the host as a signal. The host responds by strengthening the epithelial barrier, reducing inflammation, and increasing the production of mucins and antimicrobial peptides. Stimulating the colonization and growth of butyrate-producing bacteria thus may help optimizing gut health. Various strategies are available to stimulate butyrate production in the distal gut. These include delivery of prebiotic substrates that are broken down by bacteria into smaller molecules which are then used by butyrate producers, a concept called cross-feeding. Xylo-oligosaccharides (XOS) are such compounds as they can be converted to lactate, which is further metabolized to butyrate. Probiotic lactic acid producers can be supplied to support the cross-feeding reactions. Direct feeding of butyrate-producing *Clostridium* cluster IV and XIVa strains are a future tool provided that large scale production of strictly anaerobic bacteria can be optimized. Current results of strategies that promote butyrate production in the gut are promising. Nevertheless, our current understanding of the intestinal ecosystem is still insufficient, and further research efforts are needed to fully exploit the capacity of these strategies.

## Introduction

For many years, broiler intestinal health was supported by the widespread use of antimicrobial growth promoters (AGPs). These AGPs are antibiotic substances that were added to the feed at subtherapeutic levels, leading to improved animal performance. In the European Union, the use of AGPs was banned in 2006 while the Center for Veterinary Medicine of the US Food and Drug Administration, in 2012, wrote a “Guidance for Industry” document recommending that antibiotics should only be used in case of specific diseases and not for growth promotion. The most important reason for these precautionary measures was the likelihood that AGPs may increase antimicrobial resistance in bacteria, in some cases followed by introduction of these bacteria in humans (1). Most likely intestinal health problems in the chicken have been masked partly by the routine use of AGPs. Since the ban of AGPs in Europe alternative strategies for control of intestinal health have become a focus of research. These research efforts have revealed the complexity of the intestinal ecosystem. The gut indeed is an organ that contains many different cell types and exerts many different functions (2). In addition, the composition of the gut microbiota is diverse and varies depending on different factors, including the feed composition. The interaction between the host and the beneficial microbes in the intestine is mutualistic, as the host provides an anaerobic shelter and nutrients for the bacteria, which in turn provide enzymes that help digesting polysaccharides and other complex molecules (3). Nonetheless, the intestinal tract also contains facultative pathogenic microorganisms, which may be harmful and even life-threatening to the host under certain conditions. The challenge for the intestinal immune system is thus to simultaneously create a firm barrier and a powerful defense against the pathogenic bacteria, while at the same time, foster the beneficial bacteria and create an open entrance for the nutrients. These apparently conflicting tasks are taken up by the intestinal mucosa forming the so-called intestinal barrier that receives signals from the intestinal bacteria (4). Microbial signals can originate from cell wall components, like lipopolysaccharides (LPS) and flagellin, DNA fragments, and metabolites such as short-chain fatty acids (SCFAs). Those signals can have proinflammatory or anti-inflammatory effects, depending on the signal (5). One important signal derived from the beneficial microbes is butyrate.

When considering signals derived from bacterial fermentation, butyric acid has been shown to be the main driving force toward an optimal gut health. The molecule exerts beneficial effects in the physiological concentration range (6, 7). Numerous butyrate products are available on the market in a wide range of formulations. However, another possibility to increase butyric acid in the gut is by stimulating the endogenous production of butyrate by the gut microbiota. This review focuses on the methods on how to increase the endogenous production of butyrate in the intestinal tract of broilers in order to improve gut health.

## Role of the Intestinal Barrier in Gut Health

The luminal side of the intestinal wall is lined with absorptive epithelial cells, needed for water and nutrient uptake, which form a semipermeable barrier between the outside world (the gut lumen) and the internal host tissues. This semipermeable barrier is not only formed by the cell membranes of the epithelial cells but also by tight junctions that connect neighboring epithelial cells (8). These connections are regulated at different levels (e.g., by cytokines). The permeability of the intestinal epithelial cell layer can be affected by epithelial cell death and also by luminal signals that increase the epithelial layer permeability by affecting the tight junctions and thus cause loss of integrity of this important barrier (4). This loss of integrity can have detrimental effects. First, it causes an efflux of host proteins (“leaky gut”) into the lumen and can cause luminal molecules, including toxins, and microorganisms, to reach the basal side of the epithelial layer. Through coevolution, the host has developed the capacity to become tolerant to most bacteria present in the lumen at the apical side of the epithelial cells. However, bacterial components that closely interact with epithelial cells from the basolateral side do trigger an inflammatory response. This is mediated by the binding of pathogen-associated molecular patterns, such as LPS and flagellin, to the so-called Toll-like receptors that trigger the cascade of proinflammatory signals (9). It is evident that the loss of intestinal epithelial integrity and its consequences will have an effect on animal performance.

In addition to the absorptive epithelial cells, other cell types are present in the lining of the gut wall. These include mucin-producing goblet cells and antimicrobial peptide (AMP)-producing paneth cells in the crypts (10). Both cell types are very important in innate defenses. Enteroendocrine cells can also be found in the epithelial lining, secreting peptide hormones into the bloodstream. These peptide hormones have a variety of functions, including effects on epithelial cell proliferation, inflammation, and consequently intestinal barrier integrity. One of these hormones is glucagon-like peptide-2 (GLP-2), a hormone that is important in maintaining epithelial integrity (11). The enteroendocrine cells are activated by various luminal signals, including bacterial signals (see below).

Underneath the epithelial lining, many other cell types are present which together form the lamina propria of the intestinal mucosa. These cells include, among others, fibroblasts and endothelial cells. Immune cells are also present in the mucosa, both in organized lymphoid tissues and scattered between the other cells. All non-epithelial cell types are responsive to luminal triggers, whether transmitted by epithelial cell signals, including enteroendocrine cell hormones, or not, and thus can influence other cell types by themselves. Thus, intestinal barrier integrity, inflammation, and gut function are influenced by luminal signals, many of which are produced by the microbiota (12). The composition of the microbiota and the metabolites the bacteria produce are hence crucial for gut health.

## Effects of Butyrate

One of the major terminal metabolites of intestinal bacteria is butyrate. The effects of butyrate on intestinal health have already been described in detail in many reports (6, 13). The main targets of butyrate are briefly described in the following paragraph. Supplementation of butyrate in the feed can beneficially influence growth performance and intestinal villus structure in broiler chickens (14). Presence of butyrate in the intestine plays a role in the control of pathogens such as *Salmonella* Enteritidis and *Clostridium perfringens*. Reduction of *Salmonella enterica* serovar Enteritidis colonization and shedding in chickens has been reported (15, 16), as well as a decrease of necrotic lesions induced by *C. perfringens* in the small intestine (17). In addition to the role of butyrate in growth performance and pathogen control, butyrate also has anti-inflammatory properties. One of the mechanisms is inhibition of nuclear factor-kappa B activation, resulting in decreased expression of proinflammatory cytokines (18, 19). As described above, the intestinal barrier plays an important role in maintaining intestinal health. Butyrate has an effect on several components of this barrier (13, 20), such as the tight junctions (21). Butyrate upregulates AMP-activated protein kinase, which regulates the assembly of tight junctions (22). The mucin-producing goblet cells in the small intestine and distal gastrointestinal tract are also influenced by the presence of butyrate (23, 24). Butyrate-induced expression of MUC2 genes results in secretion of mucin, a glycoprotein, which forms a protective layer on the enterocytes (23, 25). AMP-producing paneth cells in the crypts are another cell type influenced by butyrate. Expression of AMPs, such as cathelicidins and defensins, is upregulated upon supplementation of butyric acid (26, 27). Finally, it is assumed that butyrate affects enteroendocrine L-cells secreting GLP-2 (28). Beneficial effects of GLP-2 on general gut health, such as the stimulation of intestinal crypt cell proliferation and the reduction of apoptosis in the crypt compartment, have been described for several animal species (29, 30). Information about the function of GLP-2 in chickens is scarce. Honda et al. (31) reported suppressed feed intake after intracerebroventricular administration of GLP-2 in chickens, which suggests that GLP-2 plays a role in regulating appetite (31). Daily intraperitoneal injection of GLP-2 in healthy broiler chickens improves absorptive function of the small intestine and has a positive effect on growth performance (32). GLP-2 receptors are thought to be present in chicken proventriculus, duodenum, jejunum, ileum, cecum, and colon, as detected by PCR (33). L-cells containing GLP-2 are mainly found in the crypts of the distal ileum and the proximal jejunum (34). This information could prove useful in unraveling the functions of GLP-2 in birds and in finding out which physiological triggers lead to secretion of the hormone (35). In humans, it is reported that butyrate enhances GLP-2 secretion and increases GLP-2 plasma concentration (35, 36). However, studies in chickens are lacking, and further research is necessary to yield conclusive information about the effect of butyrate on the release of GLP-2 in chickens.

## The Intestinal Microbiota with Focus on Endogenous Butyrate Production

The composition of the microbiota in the gut of chickens depends on the age and the gastrointestinal segment, i.e., there is both a temporal as well as a spatial variation (37). In general, the diversity of the microbiota increases with age. The composition of microbiota differs between the different segments of the chicken gut. In general, low numbers of bacteria are found in the proximal parts of the gut (stomach, duodenum, jejunum, <10^3^ cfu/g content), while the numbers increase toward the distal ileum and ceca, the latter harboring more than 10^10^ bacteria/g of content. In addition, the diversity increases significantly toward the distal gut. While only a limited diversity can be observed in the small intestine, with lactobacilli often dominant, the ceca harbor a large number of different bacterial groups (38). The cecal ecosystem of healthy subjects is dominated by bacteria belonging to the phyla *Bacteroidetes* and *Firmicutes*, together comprising more than 80% of the microbiota. The former contains many polysaccharide-degrading bacterial species, while the latter contains a variety of bacterial families, of which the *Ruminococcaceae* (*Clostridium* cluster IV) and *Lachnospiraceae* (*Clostridium* cluster XIVa) families are capable of fermenting various substrates to butyrate. The bacterial community has the genetic potential to carry out an enormous number of physiological functions (37). They can play an important role in both immune and digestive functions. Commensal bacteria can exert anti-inflammatory properties by producing SCFA (see below). Moreover, the mucosal architecture, mucus composition, and production can be affected by the presence of the gastrointestinal bacteria (39). The number of microbial genes in the gut of a chicken exceeds the number of chicken genes. Together these form a so-called “hologenome” (40).

### Butyrate-Producing Bacteria and Bacteria That Promote/Inhibit Butyrate Production

#### Butyrate-Producing Bacteria

While a large variety of bacteria can produce acetic acid, the most important butyric acid-producing bacteria belong to a limited number of families, including the *Ruminococcaceae* and *Lachnospiraceae* (41). These families contain strictly anaerobic bacteria that are highly abundant in the gut of healthy chickens, as well as mammals. Other *Clostridium* clusters, including clusters I and XVI, contain butyrate producers, but the level of butyrate produced is much lower in comparison with strains from cluster IV and XIVa (42, 43). Individual butyrate-producing strains isolated from chicken ceca have been characterized (44, 45). In 2011, a first study specifically investigated the diversity of butyrate-producing bacteria from chicken ceca. 16S rRNA gene sequence analysis of isolates revealed members of *Clostridium* clusters IV, XIVa, XIVb, and XVI (42). When butyrate-producing bacteria are present in a sufficiently high concentration, the epithelial barrier integrity will be stronger, the epithelial cells will proliferate more and thus the villi will be longer. Moreover, inflammatory reactions will be reduced, while the stimulation of regulatory T-lymphocytes will yield a state of tolerance toward non-harmful bacteria (46). Butyrate is mainly synthesized via the reductive acetyl-coenzyme A pathway (47). Via a four-step pathway, acetyl-CoA is converted to the intermediate butyryl-CoA and further to butyrate. The final step or actual butyrate formation in its synthesis is mostly catalyzed by butyryl-CoA:acetate CoA-transferase and is used by several families of the healthy gut microbiota, dominated by *Lachnospiraceae* and *Ruminococcaceae* (48, 49). Another, but less frequently used, enzyme in the final step is butyrate kinase that produces butyrate from butyrylphosphate that is derived after phosphorylation of butyryl-CoA (7). Recently, it has been hypothesized that a propionate CoA transferase related to the enzyme of *Clostridium propionicum* is responsible for butyrate formation in members of the family *Erysipelotrichaceae* (42). In addition, three other butyrate-producing pathways using amino acids as major substrates are known. These pathways, only present in 20% of potential butyrate producers, are the lysine, 4-aminobutyrate, and glutarate pathways (47).

#### Other Microorganisms That May Interact with Butyrate Production

The variety of metabolic functions of the intestinal microbiome encompasses degradation of complex substrates (polysaccharides, proteins, and fat), fermentation of substrates to yield acidic compounds, immunomodulation, communication with other bacteria, and much more. To break down complex substrates, the bacteria form a food web, with different species and strains involved in different steps of the substrate utilization process (50). In a simplified model, complex substrates such as polysaccharides, including arabinoxylans, pectins, and cellulose, are converted to oligosaccharides by specific bacterial populations such as lactobacilli and some *Bacteroides* species, among others. These oligosaccharides [e.g., arabinoxylan-oligosaccharides (AXOS)] are then used by other bacterial groups to produce lactic acid, hydrogen, and SCFAs such as acetic, propionic, and butyric acid (41). The system in which bacteria convert substrates to products that are converted by other bacteria is called cross-feeding. This is an important mechanism, clearly showing the need of a diverse bacterial community, in order to be able to degrade substrates (51, 52). In case of dysbiosis, an unfavorable shift in the composition of the microbiota occurs, leading to deficiencies in certain crucial steps in certain pathways, yielding changes in the bacterial metabolites produced in the gut (53). Bacteria that promote butyrate production through the production of intermediate metabolites, as well as bacteria that inhibit butyrate production by competition for substrates can be present in the gut. For example, the underlying mechanism by which lactic acid bacteria (LABs) can be beneficial can be explained by the effects of butyrate, considering that the lactic acid produced by these bacteria is then further consumed by *Clostridium* cluster XIV strains to produce butyrate (48). Lactic acid is toxic by itself when present in high concentrations and only has a benefit when it is converted (54). Sulfate-reducing bacteria (SRBs) compete for lactate with butyrate-producing bacteria from *Clostridium* cluster XIVa, with the outcome of this competition being a crucial factor for gut health. Apart from this, SRBs also have to compete for hydrogen with hydrogenotrophic acetogenic and methanogenic bacteria (55). They produce hydrogen sulfide as a terminal metabolite. Hydrogen sulfide, with proven cell death-inducing effects on intestinal epithelial cells, is a clearly toxic metabolite (56). One can thus conclude that a complex interaction between different bacterial populations for specific substrates exists, and the outcome of this interaction can drive the microbiota either to produce beneficial metabolites which promote gut health, or toward production of toxic metabolites.

## Tools to Stimulate Endogenous Butyrate Production

### Probiotics

The term probiotics was first defined by FOA/WHO in 2001, and at the end of 2013, the definition was worded more grammatically correct as “live microorganisms that, when administered in adequate amounts, confer a health benefit on the host” (57). The majority of bacterial probiotics consist of LABs, mainly from the *Lactobacillus*, *Bifidobacterium*, *Enterococcus*, and *Streptococcus* genera (58, 59). As discussed above, lactic acid can be consumed by butyrate-producing bacteria to produce butyrate (48). Different types of products are currently available on the market. Beside single-strains, multi-strain products are available, as well as competitive exclusion (CE) products, which contain a freeze-dried mixture of gut content. The main purpose of CE products is replacing the natural route of microbiota succession, while probiotics enhance the functions of existing microbiota. Besides, only defined CE products can be registered for which the identities of the bacteria in the mixture are known to be safe for chickens and humans (60).

Reports of the effect of probiotics on animal performance have been published (61, 62), and studies on the beneficial effect on pathogen colonization and disease reduction are widely available (63–65). However, positive effects are not observed in all studies. One of the reasons for the inconsistency of observed beneficial effects may be that success depends partly on the conditions of the study, i.e., feed formulae and feed ingredients, management factors, and presence or absence of stress factors and challenge conditions. Indeed, unpublished data from our laboratory show that the efficacy of probiotics is highly dependent on the model used. However, based on the above described data, attempts could be made to develop probiotics that stimulate butyrate production in the intestinal tract, either directly or through cross-feeding.

#### Butyrate-Producing Bacteria as Probiotic Strains

Most butyrate-producing bacteria belong to the *Clostridium* clusters IV and XIVa (66, 67) and play an important role in maintaining a healthy gut primarily through production of butyrate (7). These bacteria are less abundant in the gut of people suffering from inflammatory bowel disease (IBD) as compared to healthy persons (68). More specifically, *Butyricicoccus ­pullicaecorum* and *Faecalibacterium prausnitzii*, species belonging to *Clostridium* cluster IV, have been shown to be less abundant in the gut of IBD patients (68, 69). Thus, oral administration of these strains as probiotics might counteract IBD inflammation. At the moment, no specific studies are available in literature that show beneficial effects of butyrate-producing bacteria from *Clostridium* clusters IV and XIVa as probiotic candidates in poultry. However, *Clostridium butyricum* from *Clostridium* cluster I, administered as a component of a tri-strain probiotic, significantly improved body weight gain and reduced feed conversion rate in broiler chickens (70). Similar results have been reported with supplementation of *C. butyricum* in the feed as single strain probiotic (71).

A major problem with the production of butyrate-producing bacteria as probiotic is the cultivation, as these microorganisms require strict anaerobic conditions (72). Another issue is that most poultry feed is pelleted, and probiotic strains are exposed to high temperatures during this process. Thus, ideally the probiotic strains should be heat-stable, for example, through spore formation (73). Unfortunately, most butyrate-producing bacteria seem to lack this characteristic.

#### Other Microorganisms as Probiotic Strains Increasing Butyrate Concentration

Instead of direct administration of butyrate-producing bacteria to the bird, attempts could be made to use strains that stimulate butyrate production by *Clostridium* cluster IV and XIVa strains. The *Clostridium* cluster XIVa strains consume lactic acid to produce butyric acid. Therefore, LABs could be useful probiotics for their property of indirect stimulation of butyrate production. This cross-feeding mechanism may explain the beneficial effects of lactobacilli and bifidobacteria (70, 74). Addition of lactobacilli in an *in vitro* fermentation system of cecal broiler content increases butyrate concentration (75). This suggests that lactobacilli, as probiotic bacteria, stimulate butyrate-producing bacteria in the chicken cecum. These effects are, however, hitherto not investigated *in vivo* in chickens.

### Prebiotics

The definition of prebiotics as proposed by Gibson et al. (76) is “Selectively fermented ingredients that allow specific changes, both in the composition and/or activity of the gastrointestinal microflora that confer benefits upon host wellbeing and health” (76). Several prebiotics induce beneficial effects on broiler performance and pathogen control (77, 78). Most published studies involve oligosaccharides, such as fructo-oligosaccharides (FOS), galacto-oligosaccharides (GOS), arabinoxylo-oligosaccharides (AXOS), xylo-oligosaccharides (XOS), and raffinose family oligosaccharides (RFOs) (79). Prebiotics are complex molecules because of the chain length, the nature of the sugar bonds, and the nature of the side chains on the saccharides. These characteristics can affect their function as prebiotic (80). Prebiotics need to be converted into metabolites by the microbiota in the gut. Because they are saccharides, their end products will be SCFAs, lactate, and gases, and thus the beneficial effect can theoretically be evaluated or predicted by analyzing these metabolites.

#### Prebiotics Used By Butyrate-Producing Bacteria and Other Microorganisms

Prebiotics that stimulate colonization and activity of butyrate-producing *Clostridium* cluster IV and XIVa populations are considered to be beneficial. For example, our group showed that XOS supplementation to a broiler diet increases the number of lactobacilli and *Clostridium* cluster XIVa strains in the distal gut, thereby stimulating the cross-feeding between both groups, so that lactate is converted into butyrate (78). Different studies report improved growth performance and an increase of *Lactobacillus* and *Bifidobacterium* bacteria in the gut of broilers after administration of oligosaccharides (81–83). After supplementation of AXOS in the diet, a decrease of *Salmonella* Enteritidis and improved feed conversion rate is reported in poultry (44, 84). Contradictory results have been published with FOS supplementation in broilers. Several studies report a decrease of pathogen colonization, i.e., *Salmonella*, *C. perfringens*, and *Escherichia coli*, in the cecum of broilers (81, 85, 86), and an increase of *Bifidobacterium* spp. and *Lactobacillus* spp. (81). Other studies in broilers report no effect at all (87, 88). These discrepancies could be explained by differences in age, diet composition, and concentration of FOS in the diet. As discussed above, it can be hypothesized that the observed beneficial effects are at least partly due to the bacteria supporting the cross-feeding leading toward production of butyrate.

## Conclusion

A large number of experimental and field trials in broilers have already been carried out, using a variety of feed additives. The most commonly measured outcome parameter is performance. Some studies were also done to determine the effect on pathogen colonization. The approach was mostly empirical and the products were therefore mainly developed without a clear understanding of the mode of action underlying the hoped-for beneficial effects. The only way to develop products with an enhanced activity as compared to the already existing products will be based on a thorough understanding of the intestinal ecosystem. Identification of the microbiota components that are crucial to gut health is ongoing and forms an essential part of the proper development of additives that support gut health (67). This needs to be done in both ways, i.e., identifying both the beneficial microbes as well as the harmful ones. Current knowledge indicates that butyrate-producing bacteria and their metabolite butyrate improve gut health in the physiological range (6). The amount of butyrate produced by the microbes in the lower intestinal tracts is impressive, considering that more than 50% of the microbiome is composed of butyrate producers (7). Deficiencies in butyrate production by the microbiota for whatever reason always seem to lead to inflammation in the intestinal tract (13). Providing a butyrate supplement in the feed only gives a partial answer to the problem, as the amount that can be added to the feed is limited. Thus, there is ample reason to try and stimulate endogenous butyrate production. This can be achieved by using feed additives, i.e., prebiotics that support the proliferation and the metabolic activities of the butyrate producers. Another possible strategy to stimulate endogenous butyrate production is by direct feeding of butyrate-producing bacteria. However, most probably a range of other metabolites exists with unknown mechanisms and influences on gut health. There is a need to investigate the specific effects of the different bacteria and metabolites that are found to play a role in gut health. Bacterial culturing is a crucial tool to foster our understanding of the intestinal ecosystem and essential in studying the effect of a specific species or strain on gut health parameters (2). Only a small minority of the microbial species residing in the gut has ever been cultured, and therefore isolation and characterization of new bacterial species from the gut will yield useful information (89).

Finally, the feed–microbiota–host interactions are extremely complex, but expertise on how to steer this interaction is growing rapidly. One way is to increase the abundance of butyrate-producing bacteria from *Clostridium* cluster IV and XIVa. In the future, many more health promoting as well as harmful bacterial groups and metabolites will doubtlessly be discovered. This will further contribute to optimizing gut health and animal performance.

## Conflict of Interest Statement

The authors declare that the research was conducted in the absence of any commercial or financial relationships that could be construed as a potential conflict of interest.
